# Mechanism of Dinitrogen Reduction in a Borylene Complex by Density Functional Theory

**DOI:** 10.1021/acs.inorgchem.5c04644

**Published:** 2026-01-08

**Authors:** Siqi Yu, Michael B. Hall

**Affiliations:** Department of Chemistry, Texas A&M University, College Station, Texas 77843-3257, United States

## Abstract

Boron-centered dinitrogen reduction is an emerging field that complements the corresponding transition-metal chemistry. Comprehensive thermodynamic and kinetic analysis by density functional theory (DFT) of the full N_2_ reduction process in a cyclic­(alkyl)­(amino)­carbene (CAAC)-stabilized diborylene N_2_ complex, ((CAAC)­(Dur)­B)_2_(μ^2^-N_2_), reveals a spontaneous process under the mild conditions employed experimentally. Geometric and natural bond orbital analyses show N–N bond weakening in the early stages of N_2_ fixation. Frontier orbital analysis rationalizes the distinct geometries of the two key intermediates. The N_2_ adduct, ((CAAC)­(Dur)­B)_2_(μ^2^-N_2_), adopts an orthogonal arrangement of the two borylene fragments, whereas the diazene species, ((CAAC)­(Dur)­B)_2_(μ^2^-N_2_H_2_), has nearly coplanar fragments and a triplet ground state. In both, strong donation from the borylenes’ highest occupied molecular orbitals (HOMOs) into the N_2_ or N_2_H_2_ π* orbitals weakens the N–N bond, while orbital-symmetry considerations dictate the observed skeletal orientations. Although experiments detect protonation exclusively at nitrogen, our calculations predict that protonation at boron is thermodynamically accessible for tri- and tetraprotonated intermediates by using stronger acids and weaker reductants. However, large kinetic barriers for B-to-N proton migration would prevent these boron-protonated isomers from contributing to productive N_2_ reduction. These insights provide clues for design principles for steering future main-group catalysts for ammonia synthesis.

## Graphical abstract

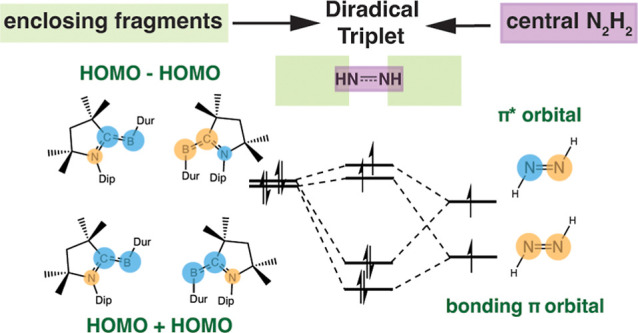

## Introduction

Ammonia is a crucial ingredient in the agricultural industry for producing fertilizers that provide food supplies for more than half of the world’s population.^1^ In addition to its chemical use, ammonia is a potential medium for high-capacity hydrogen storage^2^ and the starting material for nitrogen-based fuels that can serve as clean energy vectors.^3^ In 2023, global ammonia production was estimated at an impressive scale of around 150 million metric tons,^4^ mostly realized through the Haber–Bosch (HB) process.^5,6^ This process, the only successful dinitrogen fixation process in industry, has tremendously accelerated human productivity. Despite its irreplaceable role in industrial N_2_ fixation, HB is energy-intensive with 30% of the input energy lost to reforming processes and maintaining the high operating temperature and pressure.^7^ The process clearly leaves a large carbon footprint, which accounts for around 1.4% of worldwide greenhouse emissions.^8^

The challenge of producing ammonia in an environmentally friendly manner has attracted significant research interest. Electrocatalytic,^8^ biocatalytic,^9^ and photocatalytic^10^ synthetic systems have provided alternative pathways for N_2_ activation and reduction under ambient conditions. By far, the most efficient nitrogen fixation is the biological one catalyzed by nitrogenases, which operates under mild conditions (<40 °C, atmospheric pressure).^11–13^ The active sites of nitrogenases are composed of FeMo, FeV, or FeFe cofactors which use protons and electrons instead of molecular hydrogen.^13–15^ Inspired by the nitrogenase, complexes that utilize σ–π coordination of N_2_ by a transition metal can enable the activation and conversion of nitrogen under mild reaction conditions.^16–18^ Furthermore, metal-free complexes based on boron, such as borylene complexes studied here, are capable of emulating transition metals, facilitating σ–π bonding with N_2_ and reduction to ammonia.^19–22^ In the classical mode of N_2_ fixation at a transition-metal center, electron density flows in two directions: the unoccupied d orbitals of the metal center accept electrons from the filled σ orbital (HOMO) of N_2_, while electrons from the metal’s filled d orbitals back-donate into the vacant π* orbitals of N_2_. Both processes contribute to weakening the robust N–N bond (Figure 1a). Analogously, the empty sp^2^ orbital of boron functions like the unoccupied d orbitals of transition metals, while the filled p orbitals of boron back-donates to the N_2_ (Figure 1a).

**Figure 1:**
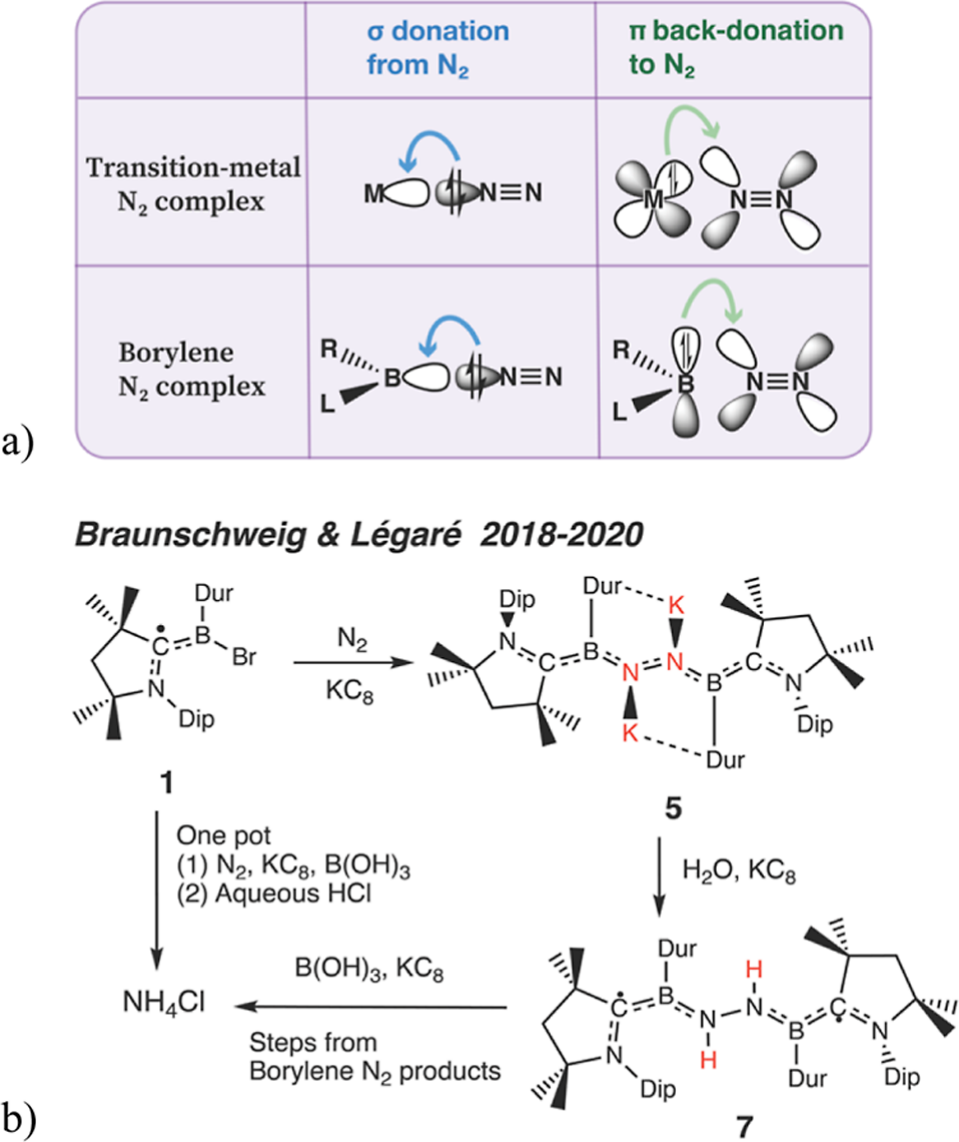
(a) Frontier-orbital analogy for N_2_ activation: both transition metal and a CAAC-stabilized borylene center interact with N_2_ through σ-donation into the empty acceptor orbital and π back-donation from a filled donor orbital into the N_2_ π* manifold. Modified with permission from Légaré et al., “Nitrogen fixation and reduction at boron,” Science, DOI: 10.1126/science.aaq1684 (2018), AAAS. (b) One-pot synthesis of NH_4_Cl from N_2_ by the dicoordinate borylene complex ((CAAC)­(Dur)­B)_2_(μ-N_2_) in the presence of KC_8_ (reductant) and B­(OH)_3_.^19,23^

Recently, Braunschweig, Légaré, and co-workers reported on a cyclic (alkyl)­(amino)­carbene (CAAC)-stabilized boron compound (Figure 1b) that enables the fixation and reduction of N_2_ at room temperature, with potassium graphite and water/boric acid as reagents.^19,23^ In this one-pot synthesis, an end-on bridging dinitrogen is converted into ammonium chloride via a stepwise reduction–protonation reaction, where a significant number of key intermediates were successfully isolated. Expanding on research within the same reaction system, Braunschweig, Holthausen, Légaré, and co-workers examined the electronic structures and magnetic properties of several intermediates and final products, demonstrating their biradicaloid character through both experimental and computational analyses.^24^ Further theoretical studies have also been published on the related reaction systems. In 2023, Guo et al. reported the synthesis of a diiminoborane compound, Mes*B≡N–N≡BMes* (Mes* = 2,4,6-tritertbutylphenyl),^20^ which, according to their report, enables the controlled release of N_2_ and can be reduced to an ammonium salt via BN triple bond cleavage. The same year, Mézailles and co-workers reported a successful N_2_ fixation using the B-centered radical anion, [Cy_2_BCl]^−^, and examined the reaction theoretically.^22^ Recently, a diboryl diazene, Mes_2_BN = NBMes_2_ (Mes = mesityl), was isolated and reported to engage ammonia and ethylamine to afford transient diazene N_2_H_2_ without radical formation.^25^

Despite extensive experimental and theoretical research on various borylene-based N_2_ fixation reactions, the insights of the details of all the steps in the N_2_ reduction processes remain limited. Addressing this gap, we conducted a detailed computational study using density functional theory (DFT), based on the experimental findings. Various proposed pathways were calculated in accordance with the conditions employed in the experimental work. A comprehensive analysis of the reaction mechanism, from both thermodynamic and kinetic perspectives, enabled us to examine the intermediates and their molecular orbital characteristics involved in the reduction process. Alternative pathways were also explored, where the N–N bond is predicted to cleave at different stages, depending on the reaction conditions, particularly by utilizing alternative proton and electron sources. Furthermore, hydrogenation at the boron atom, instead of the nitrogen, which were not observed in the experiment, was computationally investigated as potential alternative pathways.

## Computational Details

All stationary points were calculated by density functional theory (DFT) using the Gaussian 16 software package (Revision C01)^26^ with the B3LYP-D3­(BJ)^27–31^ functional. The choice of functional was based on comparisons of several alternatives (see Supporting Information Section 1 for details). Geometry optimizations were conducted with the 6-31 + G­(d) basis set^32–37^ in the gas phase. Frequency calculations at the same level were performed to calculate the free-energy thermal correction at 298.15 K (Δ*G*_corr_) and to identify any imaginary frequencies to confirm the presence of true minima (no imaginary frequency) or transition states (only one imaginary frequency). The latter were further analyzed through intrinsic reaction coordinate (IRC) calculations to confirm connectivity to the two corresponding minima. Continuum solvation model based on density (SMD)^38^ was applied to obtain solvated single-point free energies (*G*_sp_) in toluene using the optimized geometries, with the 6-311 + G­(d)^39^ basis set. Free energies in the solution phase were calculated by summing the solvated single-point energy and the above free-energy correction (*G*_soln_ = *G*_sp_ + Δ*G*_corr_). All reported free energies were referenced to the standard state conditions of 298.15 K and 1 mol/L, which requires conversion from the standard state of 1 atm to 1 mol/L, details of which are discussed in Supporting Information Section 2. Natural Bond Orbital (NBO) analyses were calculated using NBO 7.0 integrated in Gaussian to obtain the Wiberg bond indices (WBIs) at the same level of theory utilized for the geometry optimization. Only ground states were considered during reaction unless otherwise stated. Lewis structures were generated with ChemDraw (19.1) and optimized structures were visualized by the Mercury^40^ and VESTA^41^ program.

## Results and Discussion

### Thermodynamic and Kinetic Change upon Reduction by KC_8_ and H_2_O/B­(OH)_3_

Our calculations on the N_2_ fixation and reduction processes in this system are energetically favorable in all of the stages, as depicted in Figure 2. Intermediates that have been isolated and characterized experimentally are highlighted with gray shading; their optimized geometries are provided in Section 5 of the Supporting Information. Notably, the (CAAC)­(Dur)B borylene fragments in these intermediates feature conformational variability. The orientation of the Dip group (Dip = 2,6-diisopropylphenyl) on the CAAC ring relative to the Dur group (Dur = 2,3,5,6-tetramethylphenyl) generates “Anti” and “Syn” conformers, which lead to geometric and energetic differences (see Supporting Information Section 4). All intermediates in the energy diagrams are the conformers with the lowest Gibbs free energy and are in agreement with the available crystal structures.

**Figure 2:**
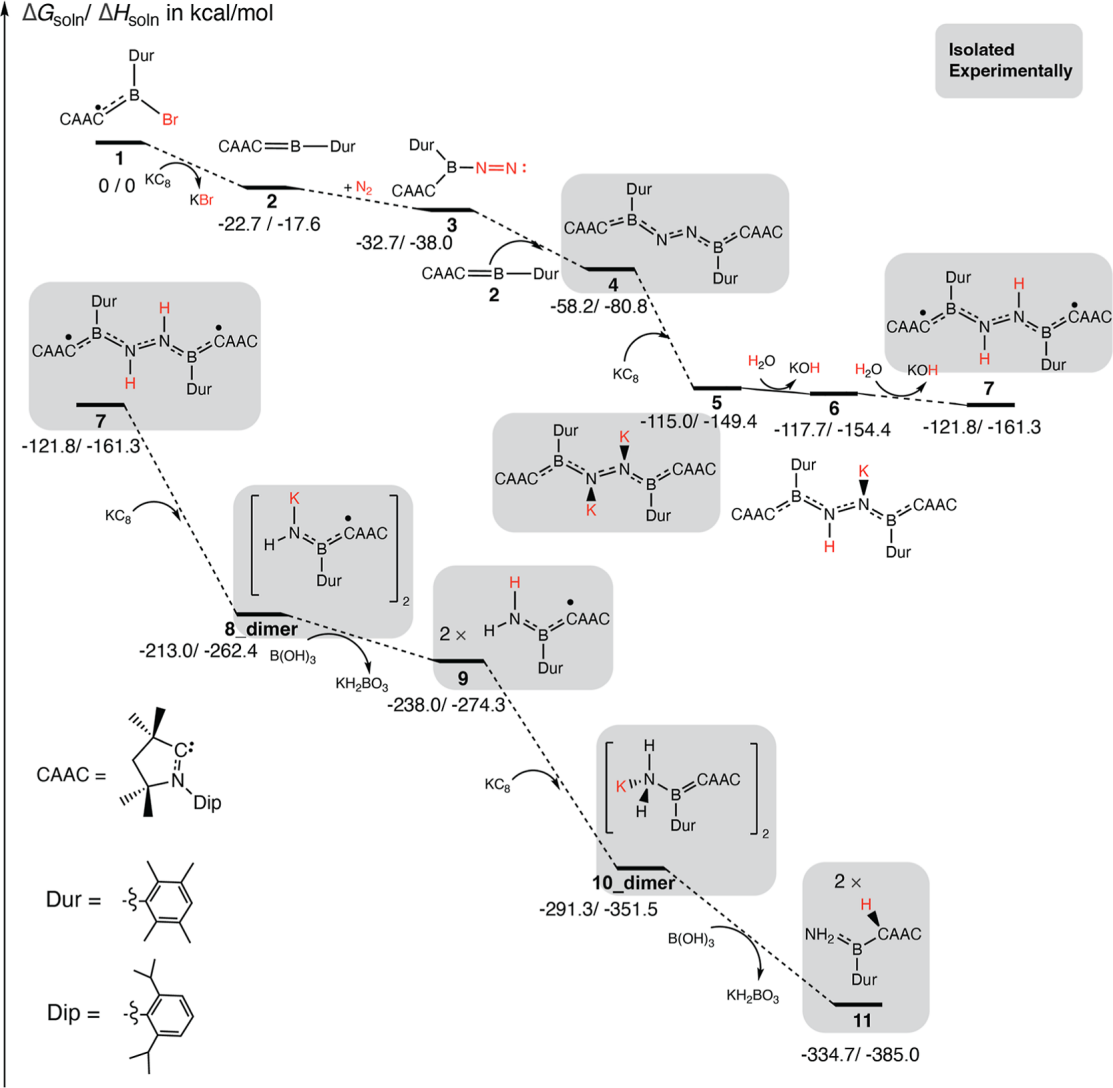
Calculated Gibbs free energy and enthalpy changes (Δ*G*_soln_/Δ*H*_soln_ in kcal/mol) in the N_2_ reduction pathway (energy not to scale) show a spontaneous reaction sequence. Initial reduction of the radical [(CAAC)­(Dur)­BBr] **1** generates borylene species **2**, two of which bind to dinitrogen to yield the dinitrogen bis­(borylene) compound **4**. Subsequent reduction and protonation steps yield **11**. Addition of concentrated HCl releases NH_4_Cl and an incompletely characterized boron-containing mixture from **11**. Species **8_dimer** and **10_dimer** (structures in Section 5, Supporting Information) are calculated as K-bridged dimers, in agreement with the experiment. Species highlighted in gray boxes have been isolated experimentally.

Specifically, upon the addition of the reductant KC_8_, boryl radical **1** loses its bromine and forms borylene species **2**, resulting in a Gibbs free-energy (*G*) decrease of 22.7 kcal/mol. This is followed by two coordination events, binding the first N_2_ reduces *G* by 10.0 kcal/mol and dimerizing with a second **2** decreases *G* by 25.5 kcal/mol, details including transition states (TSs) are given in Figure 3. Gärtner et al.^24^ also reported similar energy changes during the steps from **1** to **4**, which are discussed and compared with this work in Supporting Information (Table S1, Section 3). Notably, after two protonation/reduction steps from **4**, species **7** is formed, which leads to the complete cleavage of the N–N bond, yielding the radical amide K_2_[(CAAC)­(Dur)­B­(NH)]_2_ (**8_dimer**, Figure 2), The reduction–protonation sequence continues from **8_dimer** to **11**, during which there is a substantial decrease in free energy. In the experimental work, the reducing agent for the subsequent steps (from species **7** and on) was changed from H_2_O to B­(OH)_3_ to enable the isolation of intermediates in the latter steps.^23^ A subsequent acid quench (aqueous HCl) of **11** produced ammonium chloride as the final product.

**Figure 3:**
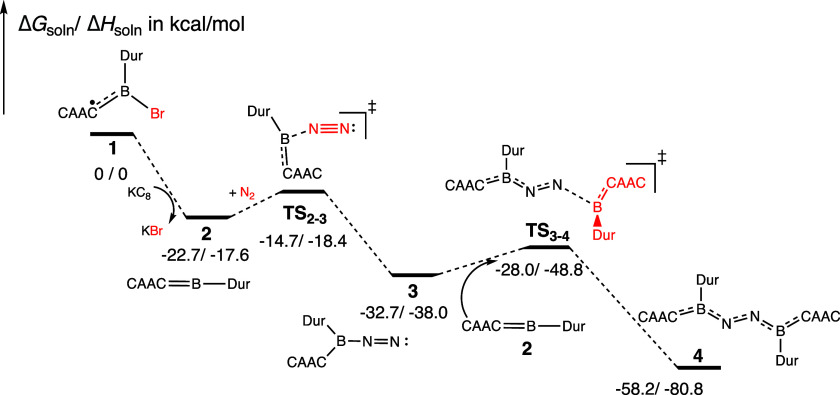
Low-energy intermediates and transition states involved in the early steps of the borylene-mediated N_2_ reduction reaction (Δ*G*_soln_/Δ*H*_soln_ in kcal/mol, energy not to scale). The free-energy barriers for the formation of the diborylene complex **4** are both less than 10 kcal/mol.

As shown in Figure 3, for the early phase of the reaction, up to bis-borylene-dinitrogen adduct **4**, all free-energy barriers are less than 10 kcal/mol. The highest barrier encountered (mainly a diffusion/entropic one) is the direct N_2_ binding to the boron site of **2** to give intermediate **3** (Δ*G* ^‡^ = 8.0 kcal/mol), which features an end-on N_2_ interaction with the borylene fragment (Figure 3). A comparable barrier of 13.0 kcal/mol at the M06-2X-D3/6-311G­(d,p) level was reported by Cummins, Gilliard, and co-workers.^42^ In the study by Gärtner et al., other interaction modes were examined, including the side-on binding, but these other modes appeared to be less favorable compared to the end-on binding modes, both kinetically and thermodynamically.^24^ Once the end-on structure is established in **3**, the subsequent reaction of another molecule of **2** at the opposite end of N_2_ to give **4** is kinetically very facile, with a barrier (Δ*G* ^‡^) of 4.7 kcal/mol. Our theoretical results indicate that both N_2_ coordination steps are fast, a result that aligns with the fact that intermediate **3** remains experimentally elusive. The N–N distance is 1.14 Å in **3**, slightly longer than that in free N_2_ (1.10 Å). For comparison, related isolable borylene-N_2_ adducts (diazoboranes) of the form RR’BN_2_ show N–N distances with a range of 1.141 to 1.160 Å.^42–44^ Along the reduction sequence, the N–N bond further lengthens to 1.24 Å in **4**, close to that for a typical N=N bond (Table 1).

**Table 1:** Calculated N–N Bond Distance Change upon Reduction

species	structure	calculated N–N bond distance in Å	experimental NN bond distance in Å	calculated NN WBI
**free N**_**2**_ **H**_**4**_	N_2_H_4_	1.42^b^	1.45 ^46^	1.06
**free N**_**2**_ **H**_**2**_	N_2_H_2_	1.22^b^	1.25 ^46^	2.13
**free N**_**2**_	N_2_	1.10	1.10 ^46^	3.03
**TS**_**2**__–__**3**_		1.11	---^a^	2.90
3	(CAAC)(Dur)B(N_2_)	1.14	---^a^	2.64
**TS**_**3**__–__**4**_		1.14	---^a^	2.40
**4**	((CAAC)(Dur)B)_2_(N_2_)	1.24	1.25	1.57
**5**	K_2_[((CAAC)(Dur)B)_2_(N_2_)]	**1**.**30** (singlet),^c^ 1.40 (triplet)	1.30 (singlet)	**1**.**56** (singlet),^c^ 0.65 (triplet)
**TS**_**5**__–__**6**_		1.31 (singlet), **1**.**40** (triplet)^c^	---^a^	1.46 (singlet), **0**.**62** (triplet)^c^
6	K[((CAAC)(Dur)B)_2_(N_2_H_2_)]	1.32 (singlet), **1**.**40** (triplet)^c^	---^a^	1.30 (singlet), **0**.**57** (triplet)^c^
**TS**_**6**__–__**7**_		1.36 (singlet), **1**.**42** (triplet)^c^	---^a^	1.15 (singlet), **0**.**53** (triplet)^c^
**7**	((CAAC)(Dur)B)_2_(N_2_H_2_)	1.36 (singlet), **1**.**40** (triplet)^c^	1.40 (triplet)	1.12 (singlet), **0**.**52** (triplet)^c^

a Not applicable.

b For reference, a typical N–N covalent single bond length is 1.42 Å,^45^ whereas a typical N=N double bond is 1.24 Å.^46^

c Values corresponding to each species’ ground state are shown in **bold**.

The reduction of **4** by KC_8_ is a highly exergonic process that leads to the formation of **5**, which has two K^+^ cations closely associated with the nitrogens. This doubly reduced species **5** has a singlet–triplet gap of 9.2 kcal/mol, which decreases to 2.3 kcal/mol upon interaction with water, forming intermediate **5**_**H2O**_ (Figure 4). The singlet and triplet transition states for the first protonation step (^**1**^ **TS**_**5**–**6**_ and ^**3**^ **TS**_**5**–**6**_) were calculated to be nearly equal in free energy (Figure 4). Despite their similar energies, their N–N bond distances differ significantly with the singlet at 1.30 Å and the triplet at 1.41 Å. The product of the first proton transfer, **6** with a NK-NH structure, is calculated to be a triplet state, but by only 0.6 kcal/mol (Δ*G*). The triplet ground state is maintained from **6** through **7**, with the singlet–triplet gap increasing to 9.4 kcal/mol for **7**, in agreement with electron paramagnetic resonance (EPR) findings for species **7**.^19^ Although the minimum energy crossing point (MECP) could not be located, the energy barriers calculated for each state on their respective potential energy surface (PES) are remarkably small, each less than 2 kcal/mol. The N–N bond distance remains around 1.40 Å from **6** to **7**, which is close to a N–N single bond (1.42 Å),^45^ until bond breakage occurs upon the reduction of **7**.

**Figure 4:**
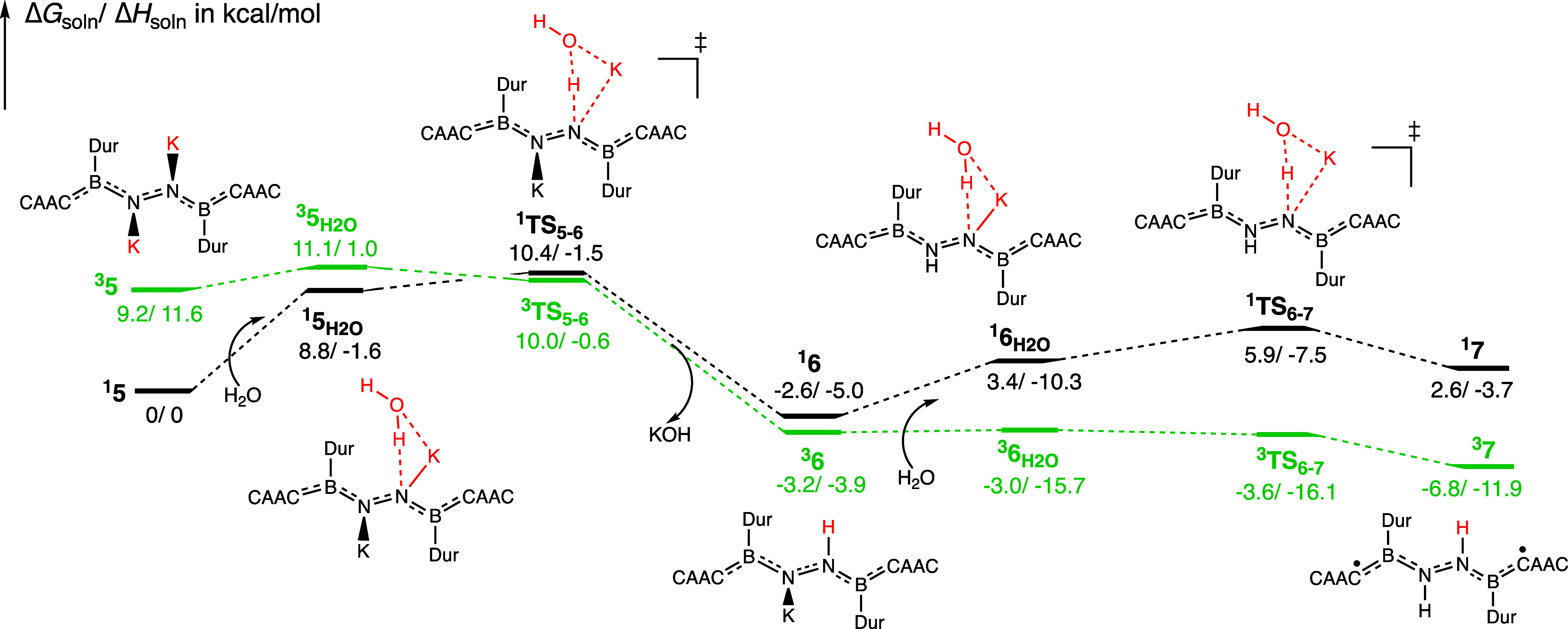
Calculated Gibbs free energy and enthalpy (Δ*G*_soln_/Δ*H*_soln_ in kcal/mol) of intermediates and transition states associated with the spin-state crossing step from **5** to **7** (energy not to scale) are shown in black for the singlets and in green for the triplets. Complex **5** has a singlet ground state with a singlet–triplet gap of 9.2 kcal/mol. The state crossing appears to occur during the first protonation step (**5** → **6**), yet a small singlet–triplet gap (0.6 kcal/mol) in **6** leaves open the possibility of a later spin crossing. The triplet-state assignment for **7** agrees with the experimental work. The slight negative barriers in both Δ*G*_soln_ and Δ*H*_soln_ from **6_H__2O_** to **^3^TS_6–7_** could be due to gas-phase geometry optimization and basis set superposition error (BSSE).

In subsequent experimental steps, B­(OH)_3_ was used instead of water to facilitate the isolation of the intermediates. From species **8** to **9**, the transition state (**TS**_**8**–**9**_) was calculated to be nearly the same energy as reactant **8** (Figure 5), suggesting a rapid hydrogen transfer from the boric acid, indicative of this flat energy surface. For the conversion from intermediate **10**_**Boric**_, where boric acid is weakly connected to the C^CAAC^ of **10**, to the transition state **TS**_**10**–**11**_, the energy barrier is 21.9 kcal/mol.

**Figure 5:**
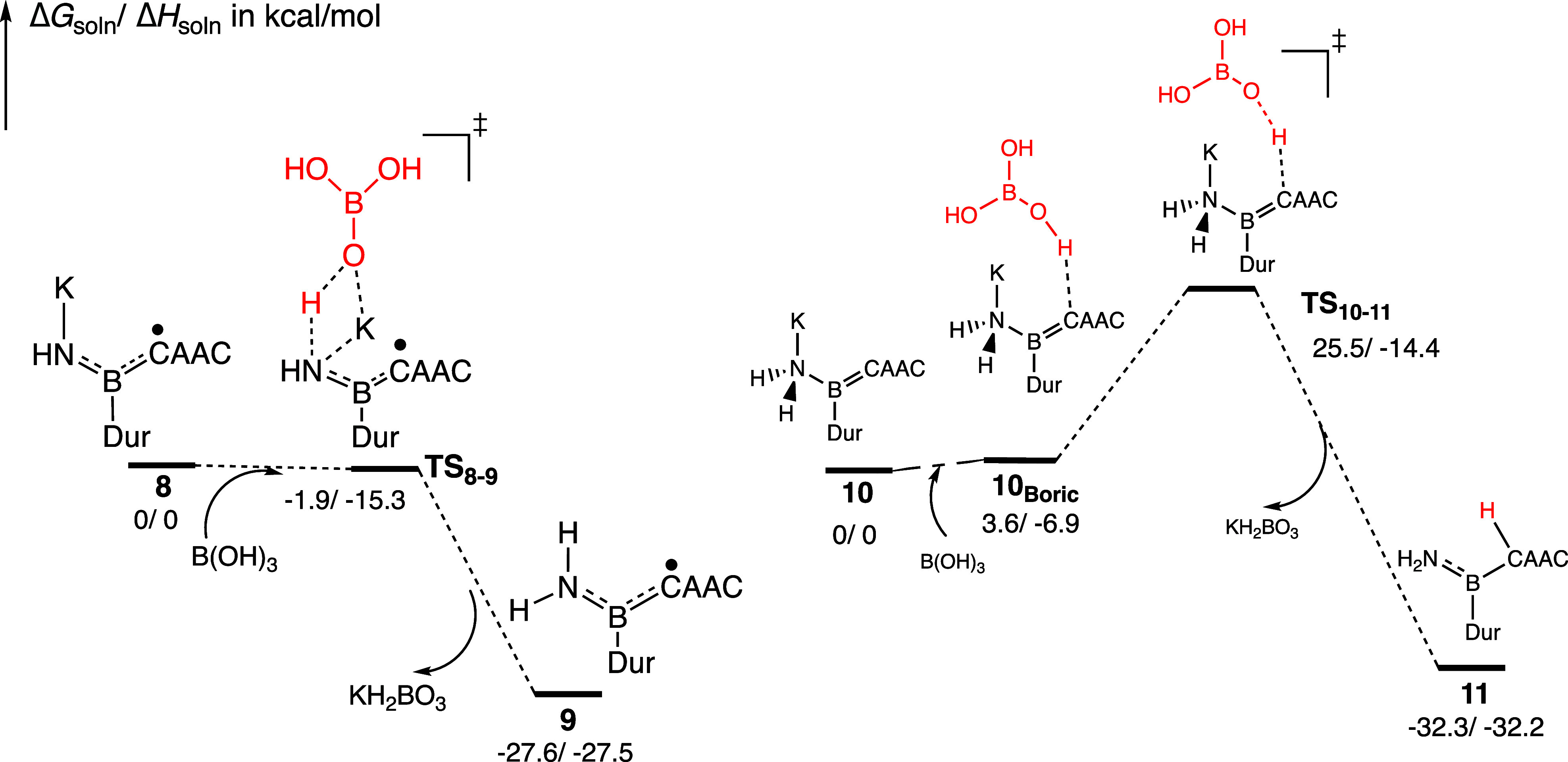
Intermediates and transition states are mediated by boric acid during the protonation steps from **8** to **9** (left), and from **10** to **11** (right) (Δ*G*_soln_/Δ*H*_soln_ in kcal/mol, energy not to scale). The slight negative free-energy barrier from **8** to **9** may be due to the gas-phase geometry optimization and BSSE and neglect of the diffusion barrier. In the second reaction, replacing boric acid with the weaker acid, H_2_O, increases the barrier from 25.5 to 35.7 kcal/mol.

### The Nature of the Borylene Complexes

To rationalize the N–N bond weakening along the course of the reaction, we investigated the electronic structure of the π-system orbital for key species such as **3**, **4**, and **7**. The structure of **3** displays a nearly planar NCBN_2_ backbone (Figure 6), which allows strong out-of-plane π bonding between its molecular components. The highest occupied molecular orbital (HOMO) of the [(CAAC)­(Dur)­B] borylene fragment, primarily composed of boron p orbital, bonding to the C of the carbene and antibonding to its N π orbital, interacts with the unoccupied π* orbital of the end-on N_2_ (Figure 6). This interaction results in the delocalization of these two electrons into the antibonding LUMO complex of N_2_. This initial phase of N_2_ activation is relatively weak, evidenced by the minor elongation of the N–N bond length by 0.04 Å from that of free N_2_.

**Figure 6:**
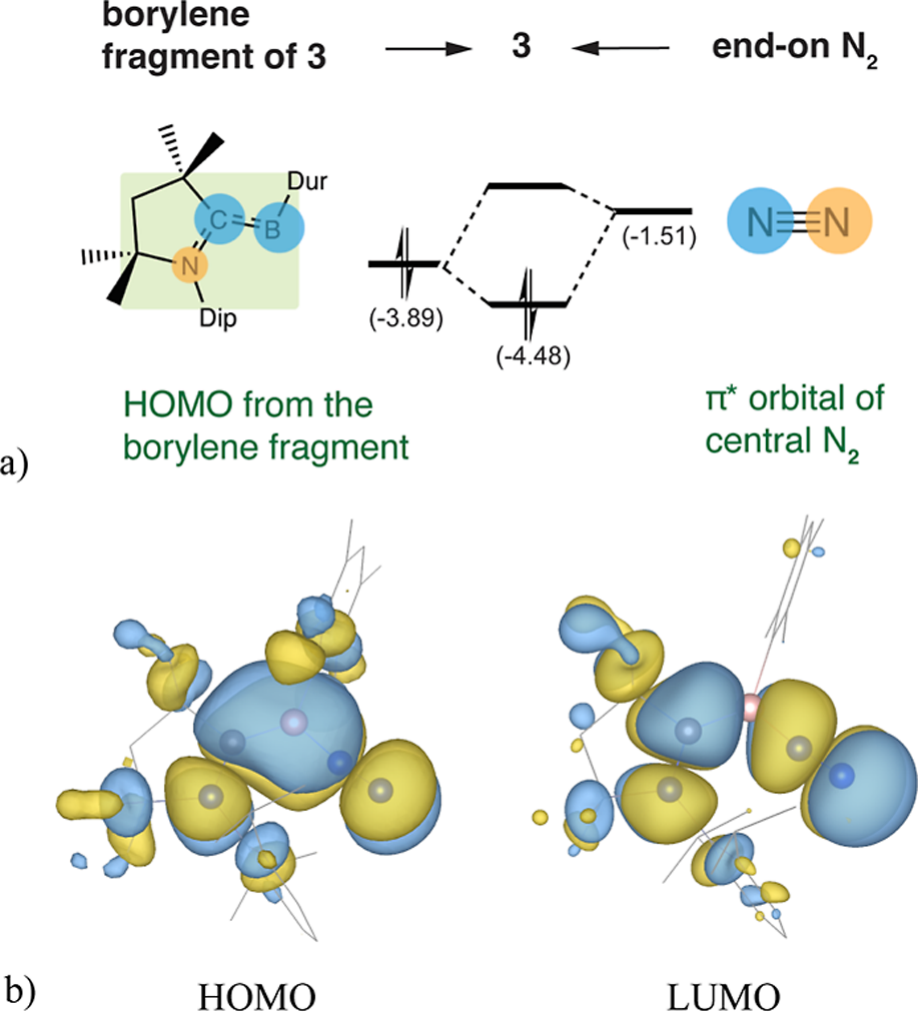
(a) The top view for the π-system and the orbital correlation diagram (energies in eV) for the formation of [(CAAC)­(Dur)­B­(N_2_)] (**3**) shows an end-on N_2_ interacting with a borylene fragment [(CAAC)­(Dur)­B], where the backbone is nearly planar (green plane). (b) The resulting frontier molecular orbitals of **3**, optimized at the B3LYP-(D3)­BJ/6-31 + g­(d) level (isovalue = 0.02, hydrogens omitted for clarity), show strong delocalization of the C–B π bond into the π* orbital of N_2_.

The formation of complex **4** involves the second [(CAAC)­(Dur)­B] borylene fragment’s HOMO interacting with the other N_2_ π* orbital, which lies in the perpendicular plane. Figure 7 presents the qualitative MO correlation diagram for this system. The HOMO and HOMO–1 of **4** arise from the interaction between the two borylene fragments’ out-of-plane π orbitals (analogous to those in complex **3**) with each of the central N_2_ two π* orbitals. The near-perpendicular orientation of the two sides of **4** (B–N–N–B dihedral angle of 110.7°) can be rationalized by the inherently perpendicular degenerate π* orbitals of central N_2_. As a result, two low-lying MOs are formed, both featuring a nodal plane at the center of the N–N bond (Figure 7b). The electron density transfer from the borylene donor orbitals to the central N_2_ π* antibonding orbitals (acceptor LUMO) consequently weakens the N–N bond and elongates it by 0.15 Å (WBI is lowered from 3.03 in free N_2_ to 1.57, Table 1). The deviation of the torsion angle from 90° results from steric hindrance in the bulky molecular structure. This interpretation is supported by calculations on a truncated model of **4** (**4_trun**), where replacement of methyl and isopropyl groups with hydrogen atoms yields a nearly perfect perpendicular arrangement between the two sides of **4_trun** (B–N–N–B dihedral angle of 88.9°).

**Figure 7:**
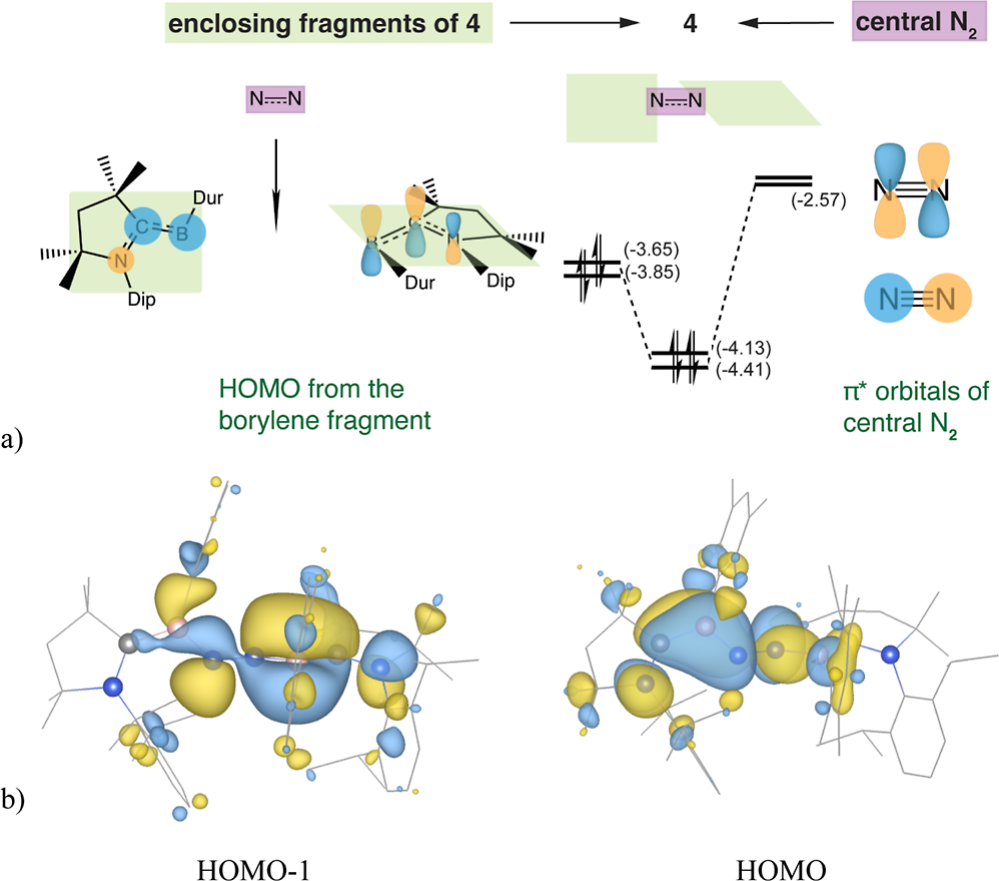
Origin of the nearly perpendicular orientation between the two borylenes of **4** lies in each one bonding to one of the two different π* orbitals of N_2_. (a) The top view of the π-system and the orbital correlation diagram (energies in eV) for complex shows the central N_2_ and two capping borylene fragments, each with nearly planar backbone oriented perpendicularly as indicated by the green planes. (b) The two frontier molecular orbitals of **4**, optimized at the B3LYP-(D3)­BJ/6-31 + g­(d) level (isovalue = 0.02, hydrogens omitted for clarity), are closely related to the corresponding orbital in **3**.

In contrast to the two nearly perpendicular sides in **4**, double reduction and double protonation results in a nearly planar NCB­(N_2_H_2_)­BCN backbone with the BNNB dihedral angle of 166.9° in **7**, as illustrated in Figure 8. Here, the HOMOs from the two planar borylene fragments result in a nearly degenerate pair of donor orbitals, namely, HOMO + HOMO and HOMO – HOMO. The latter is of appropriate symmetry to interact with vacant π* orbital (LUMO) from the central diazene (N_2_H_2_) to form the SOMO of **7**, which maximizes the orbital overlap. In addition, the HOMO + HOMO set is destabilized by the more stable π bonding orbital of N_2_H_2_, forming the SOMO–1 of **7**. Due to electron transfer from the HOMOs of the capping borylene fragments to the orbitals of the central N_2_H_2_ structure, analogous to the interactions in **4**, the N–N bond of **7** elongates to 1.40 Å as compared to 1.10 in free N_2_ (WBI is lowered from 3.03 (free N_2_) to 0.52, Table 1). The spin density of **7** is highly delocalized, as illustrated in Figure 9. Despite this delocalization, convention dictates that the “radical dots” be placed on the carbene carbons in the Lewis structures. This delocalization, consistent with the experimental EPR findings, underscores the biradicaloid nature of compound **7**. Similarly, radicals **1**, **8**, and **9** all feature pronounced delocalization of spin density across their central skeleton, as shown in Figure S6 (Supporting Information, Section 5).

**Figure 8:**
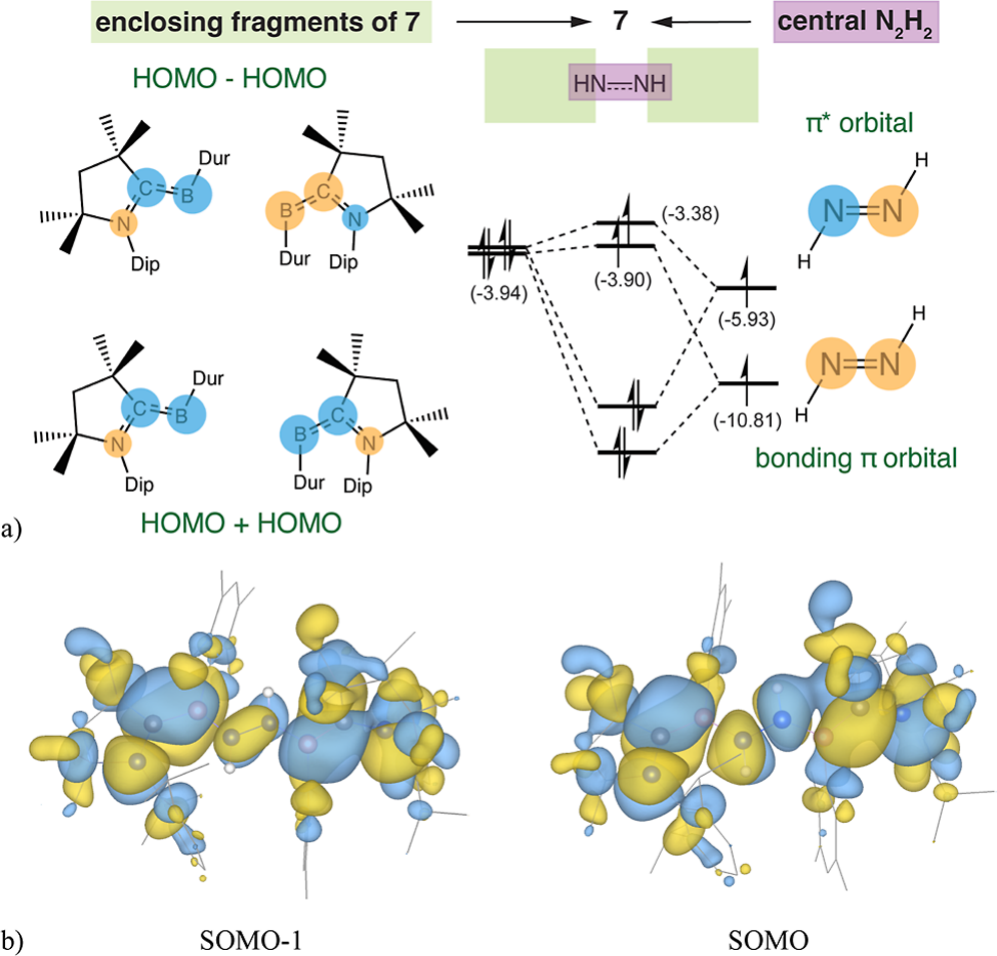
Origin of the nearly coplanar arrangement of the two borylenes of **7** lies in each one bonding to the same π* orbital of N_2_H_2_. (a) The top view of the π-system and the orbital correlation diagram (energies in eV) where the addition of 2 electrons and 2 protons to the central N_2_ from **4** results in the central N_2_H_2_ with only one vacant π* orbital for the donation of the two C–B pairs, each with a nearly planar backbone. (b) Frontier molecular orbitals of **7**, optimized at the B3LYP-(D3)­BJ/6-31 + g­(d) level (isovalue = 0.02, hydrogens omitted for clarity), illustrate the orbital symmetry in determining the observed planar orientation between the two halves of **7**.

**Figure 9:**
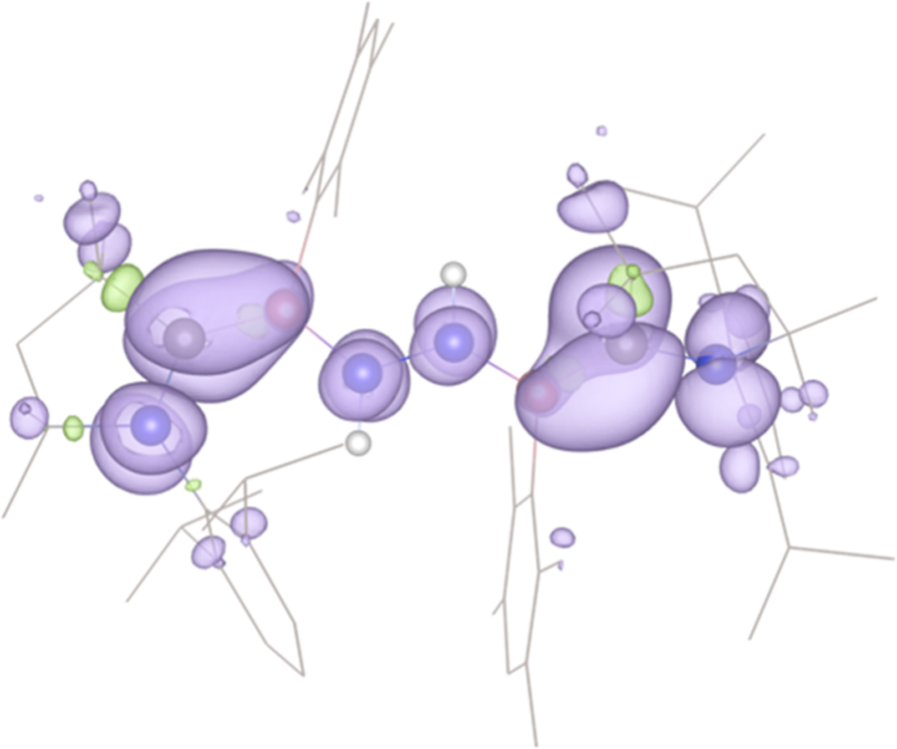
Spin density plot of **7** showing high delocalization across the core NCBN_2_BCN framework (isovalue = 0.0004, hydrogens omitted for clarity), with somewhat more spin density in the C–B bonding regions.

#### Alternative Pathway: Impact of the Acid, Reductant, and Protonation on the Mechanism

After reduction, the protonation is not confined to the bridging nitrogen atoms, as observed experimentally; our calculations suggest that protonation at the boron is also a possibility, leading to isomeric structures with even lower Gibbs free energies compared to those of their nitrogen-protonated counterparts. Figure 10 illustrates a scheme of possible intermediates and pathways within the N_2_ reduction process. The species are labeled based on the number of hydrogens they possess. For instance, the **[4H]** ^**0**^ designation encompasses neutral species that possess B**H**–N**H**–N**H**–B**H**, B**H**–N**H**–N**H**_**2**_–B, B**H**–N**H**_**2**_–N**H**–B, or B–N**H**_**2**_–N**H**_**2**_–B structure (relative energies for the alternative isomers are given in Supporting Information (Table S4) and a few key examples are given in Table 2). Horizontal arrow represents protonation, while vertical arrow indicates reduction. The strength of the proton and electron sources critically influences the reaction pathway; stronger acids and weaker reductants steer the reaction toward alternative routes with more cationic intermediates (the blue route in Figure 10).

**Figure 10:**
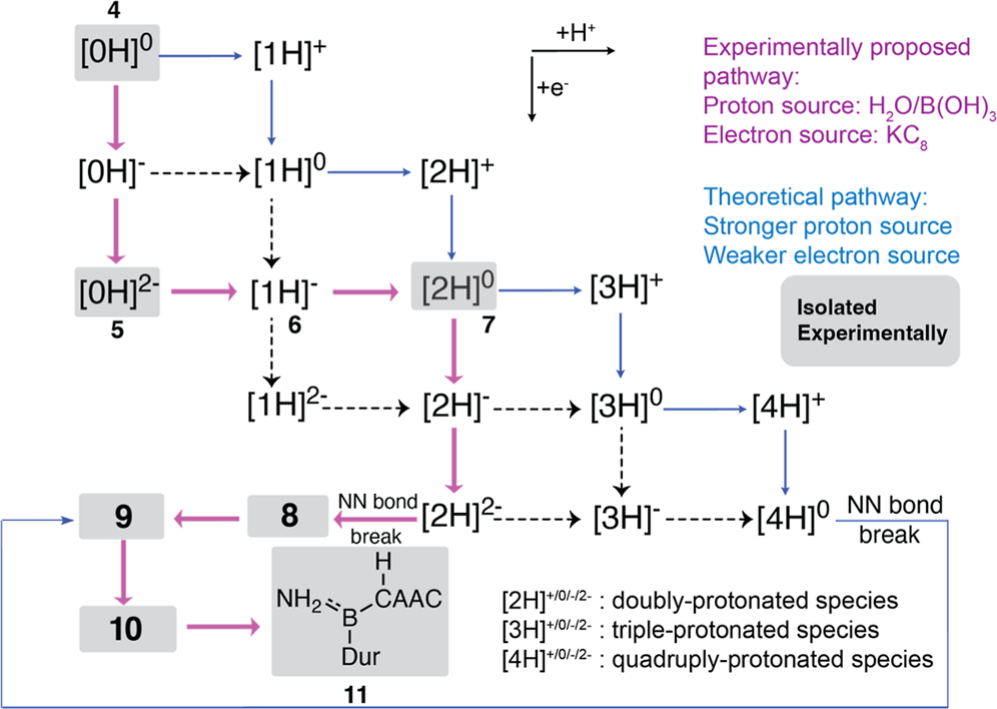
Reaction pathway for the N_2_ reduction mechanism depends on the nature of the reductant and acid. The experimentally observed pathway is denoted by bold purple arrows, while an alternative pathway using a stronger acid and weaker reductant is depicted by smaller blue arrows. In the latter pathway, the N–N bond remains intact until the formation of the NH_2_NH_2_ structure. Species are labeled based on their protonation state; for example, [2H]^2–^ is a dianion with two hydrogen atoms, which may exist as B–N**H**N**H**–B, B**H**–N**H**–N–B, or B–N**H**_**2**_–N–B structures. Species in gray boxes have been isolated experimentally.

**Table 2:** Electronic Energy Differences for Triply and Quadruply Protonated Species

species	critical structure	relative electronic energy (Δ*E*_e_, kcal/mol)
**[3H]** ^+^	BH–NH–NH	0.0
	NH_2_–NH	19.0
**[3H]** ^**0**^	BH–NH–NH	0.0
	NH_2_–NH	12.1
**[3H]** ^–^	BH–NH–NH	6.6
	NH_2_–NH	0.0
**[4H]** ^+^	NH_2_–NH_2_	89.0
	BH–NH–NH_2_	55.4
	BH–NH–NH–BH	48.2
	BH–NH_2_–NH	37.7
	NH_2_···NH_2_ (N–N bond breaks)	0.0
**[4H]** ^**0**^	BH–NH–NH–BH	62.7
	BH–NH–NH_2_	58.7
	BH–NH_2_–NH	52.9
	NH_2_···NH_2_ (NN bond breaks)	0.0

For species that are singly and doubly protonated (**[1H]** ^+/**0**/‑/**2**‑^ and **[2H]** ^+/**0**/‑/**2**‑^), those with hydrogen on the boron atom possess significantly higher electronic energies (by more than 15 kcal/mol) compared to their most stable counterparts (Table S4). In contrast, for triply protonated species (**[3H]** ^+/**0**^), isomers with hydrogen atoms on their boron atoms, such as [BH–NH–NH]^+/0^, are energetically favored over their [B–NH_2_–NH]^+/0^ counterparts by 19.0 and 12.1 kcal/mol for cationic and neutral species, respectively (Table 2 and S4). The triply and quadruply protonated species are predicted only for the cases with stronger acids and weaker reductants. Interestingly, hydrogen migration from boron to nitrogen sites is predicted to have high activation barriers (detailed in Supporting Information, Section 4.3).

Experimentally, the reduction of **7** (with an NH–NH structure) results in the cleavage of the N–N bond. However, by using a stronger acid and weaker reductant, the N–N bond can be preserved during further reduction until the NH_2_–NH_2_ structure forms. Among the four isomers of **[4H]** ^**0**^, like those of **[3H]** ^**0**^, hydrogenation on boron proves to be more thermodynamically favorable than that on the central nitrogen (Table S4). Nevertheless, subsequent N–N bond cleavage upon further reduction significantly lowers the free energy, driving the reaction in this direction.

## Conclusions

We have analyzed the key kinetic and thermodynamic steps in the N_2_ reduction reaction by this diborylene complex, confirming its spontaneous nature. The reduction process primarily weakens the N–N bond in N_2_ by binding to the second borylene and forming the diborylene complex **4** (**T**_**3**–**4**_ to **4**) and the subsequent initial hydrogenation step (**5** to **6**), as substantiated by NBO analysis. The orbital symmetry, as demonstrated using both full and truncated models, elucidates the near-perpendicular orientation of the two halves in **4** and their nearly planar configuration in **7**. These geometric preferences generate significant N_2_ activation, driven by electron donation from the HOMOs of the borylene fragments into the π* antibonding orbitals of N_2_. Experimental results indicate that the N–N bond breaks upon reduction of the NH–NH structure; however, the use of stronger acids and weaker reductants could preserve this bond until the NH_2_–NH_2_ structure forms. Although alternative hydrogenation at boron sites is thermodynamically favorable, it is limited in N_2_ reduction reactions due to significant energy barriers impeding hydrogen transfer from boron to nitrogen sites. Our study suggests that selecting the appropriate proton and electron sources may not only suppress undesired side reactions but also enable more efficient or mechanistically distinct N_2_ reduction pathways.

## Supplementary Material





## Notes

Computational methods and benchmarking; structural details and spin-density plots; and additional figures/tables (PDF)
Optimized Cartesian coordinates for all species (XYZ)
