# Supplementary material for: Mechanism of Dinitrogen Reduction in a Borylene Complex by Density Functional Theory
Source: Inorg Chem. 2026 Jan 8;65(17):9318–26. doi: 10.1021/acs.inorgchem.5c04644 (PMC13147322; doi:10.1021/acs.inorgchem.5c04644)
Supplement: Supplementary file 1 [file ic5c04644_si_001.pdf]

Supporting Information:

Mechanism of Dinitrogen Reduction by a Borylene Complex by Density Functional Theory

Siqi Yu and Michael B. Hall*

Department of Chemistry, Texas A&M University, College Station, TX 77843-3257

*To whom correspondence may be addressed. E-mail: sn72@tamu.edu and MBHall@tamu.edu

Table of Contents

1. Test of density functionals......2

2. Correction to Gibbs free energy in the solution phase......4

3. Energy change during the initial N_2 fixation from intermediates 1 to 4, a comparison with work by Gärtner et al. 5

4. The "Anti" and "Syn" structures of the borylene complexes......6

4.1. The comparison for species 4 in different conformations......8

4.2. The isomers involved for each species in the reaction......10

4.3. Isomerization of hydrogen transfer from boron to nitrogen site......14

5. Optimized structures and spin density 15

6. References......20

1. Test of density functionals

Geometry optimization in the gas phase for the species **1**, **4**, **5, 7** and **9**, with 6-31+g(d) basis set and commonly used functionals including BP86, B3LYP, ωB97XD, TPSS, TPSSH, M06, M06L, and MN15, with or without Grimme's dispersion correction. The experimental crystallographic structures were used as a benchmark to evaluate the accuracy of different functionals. For each species, the calculated bond distances of its "backbone" were compared with the experimental value and the root mean square deviation (RMSD) of those bonds were calculated for each selected functional. The "backbone" bonds are N1-C1, C1-B1, B1-N2, N2-N3, N3-B2, B2-C2, C2-N4 of **4**, **5, 7**, N1-C1, C1-B1, B1-Br of **1** and N1-C1, C1-B1, B1-N2 of **9** (Figure S1).

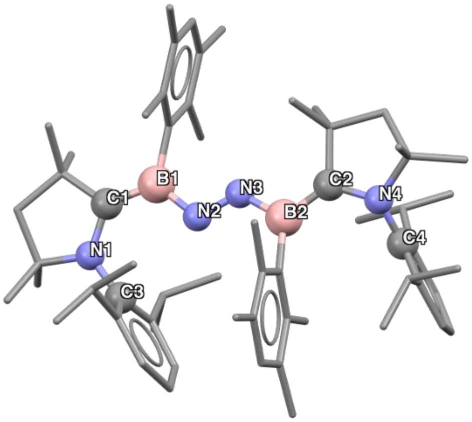

4

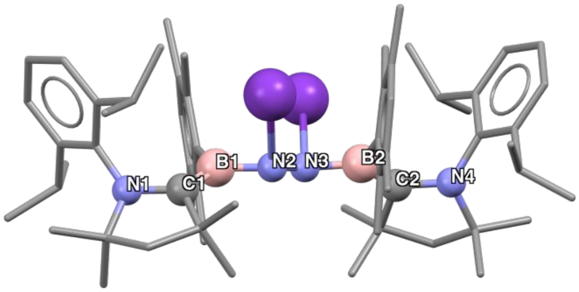

5

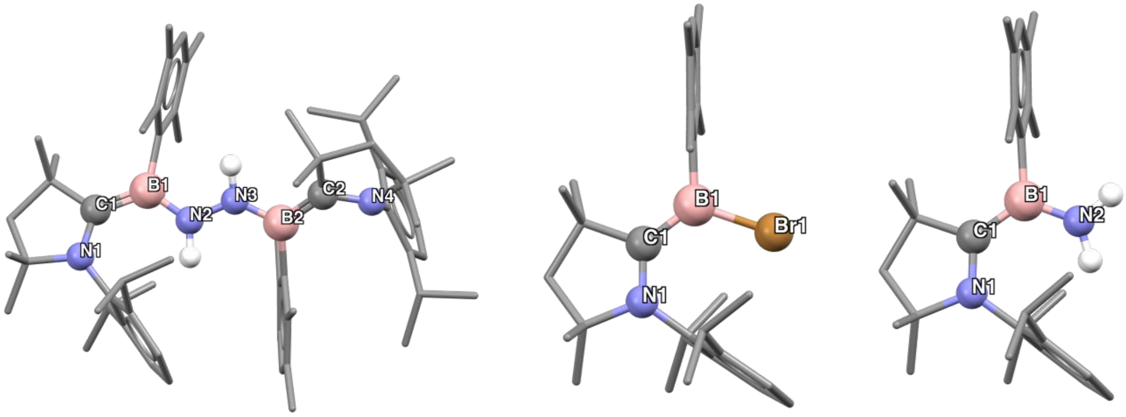

7 **1** **9**

Figure S1. DFT optimized structures of complexes **4, 5, 7, 1** and **9**, with “backbone” atoms labeled. Hydrogens are omitted unless on the N atom.

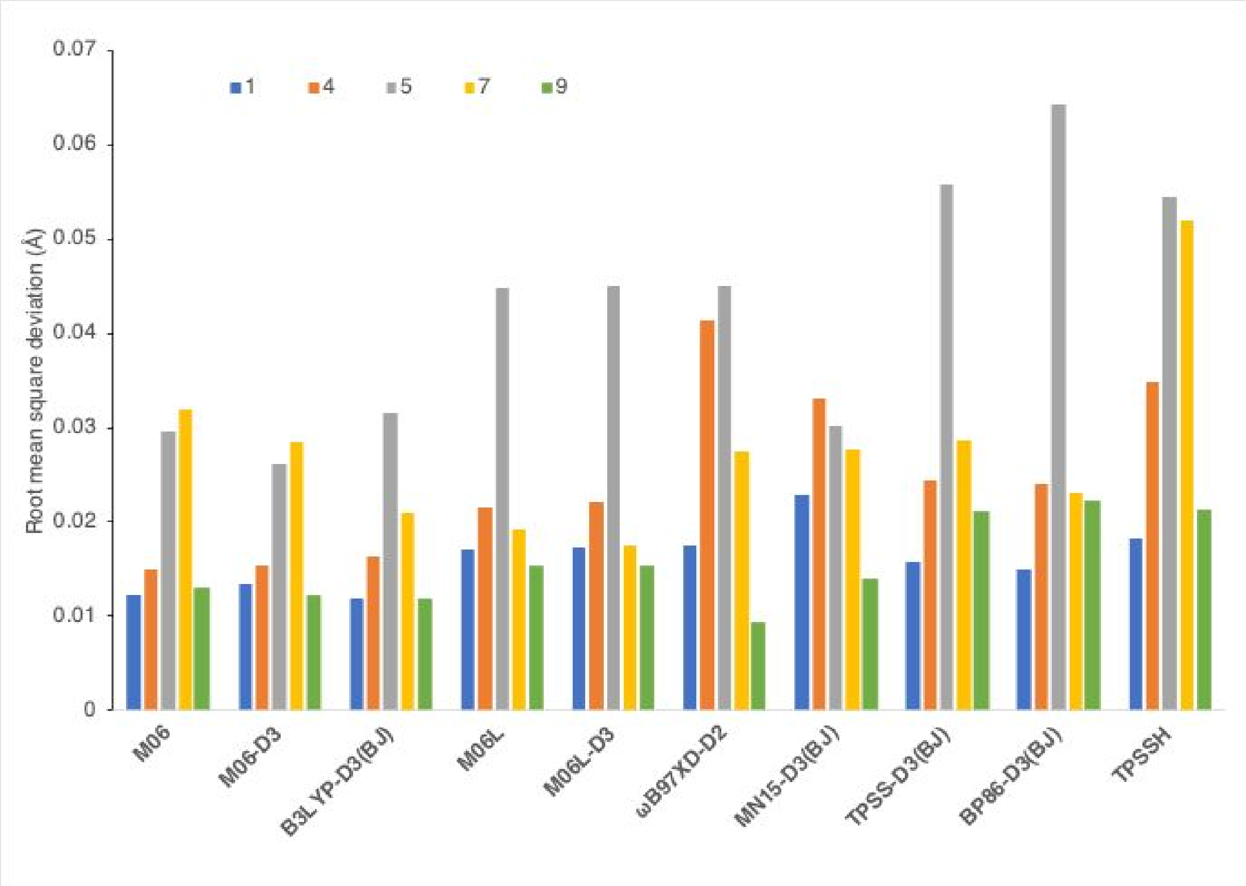

Figure S2. RMSD of the "backbone" bond distances (Å) of **4**, **5**, **7**, **1** and **9**, the main species involved in the reduction process, optimized by various functionals using 6-31+g(d) basis set.

Among all the functionals, M06, M06-D3 and B3LYP-D3(BJ) have the smallest RMSD value. Considering their performance on the most critical N-N bond distance, B3LYP-D3(BJ) was chosen for further calculation.

2. Correction to Gibbs free energy in the solution phase

In Gaussian 16, by default, the standard state Gibbs free energy is given at 298.15 K and 1 atm (denoted as G^{1atm}), even with implicit solvation model applied. In order to convert from a standard state that uses a gas-phase pressure of 1 atm to the solute standard state concentration of

1mol/L (standard molar Gibbs free energy, denoted as G^{1M}), 1.89 kcal/mol [1] has to be added ($G^{1M} = G^{1atm} + 1.89\ kcal/mol$).

For a reaction that has no molar change, consider the protonation step of formation of **7** from **5**:

$K_2[\{(CAAC)(Dur)B\}_2(N_2)] + 2H_2O \rightarrow [\{(CAAC)(Dur)B\}_2(N_2H_2)] + 2KOH$

(**5** + $2H_2O \rightarrow$ **7** + 2KOH)

The standard conversion (from 1 atm to 1 mol/L) cancels itself on both sides of reaction because the sums of reactants molarities and products molarities are the same,

$G_7^{1M} = G_7^{1atm} + 1.89$ (similar for **5**, H_2O and KOH)

$$\Delta G^{1M} = G_7^{1M} + 2G_{KOH}^{1M} - G_5^{1M} - 2G_{H2O}^{1M} = (G_7^{1atm} + 1.89) + 2(G_{KOH}^{1atm} + 1.89) - (G_5^{1atm} + 1.89) - 2(G_{H2O}^{1atm} + 1.89) = G_7^{1atm} + 2G_{KOH}^{1atm} - G_5^{1atm} - 2G_{H2O}^{1atm} = \Delta G^{1atm}$$

thus ΔG stays the same. ($\Delta G^{1M} = \Delta G^{1atm}$)

But for a reaction that changes in molar number, consider the associate reaction of [(CAAC)(Dur)B] (species **2**) with N_2 to form the adduct:

$[(CAAC)(Dur)B] + N_2 \rightarrow [(CAAC)(Dur)B(N_2)]$

The ΔG changes due to unequal molarities on two sides of the reaction, $\Delta G^{1M} = \Delta G^{1atm} - 1.89$. Similar for other reactions that involves change in molar number.

3. Energy change during the initial N_2 fixation from intermediates 1 to 4, a comparison with work by Gärtner et al.

Table S1. Calculated energy change during the initial N_2 fixation from **1** to **4**.

Relative Electronic Energy (E_e) in kcal/mol			Relative Gibbs Free Energy (*G*) in kcal/mol	
**Methods**	**B3LYP-D3(BJ)/ 6-31+G(d)**	**M062X-D3/ 6-31+G(d,p)**	**SMD(Toluene)-B3LYP-D3(BJ)/6-311+G(d)**	**SMD(Et_2O)-M062X-D3/ def2-TZVP**
**Species**	**This work**	**Gärtner et al**	**This work**	**Gärtner et al**
**1**	---	---	22.7	---
**2**	---	---	0	0
**TS_{2-3}**	22.8	18.9	8.0	10.9
**3**	0	0	-10.0	-5.4
**TS_{3-4}**	-15.9	-17.0	-5.3	5.7
**4**	-51.6	-51.8	-35.5	-23.9

Relative Electronic Energy (E_e) are similar and comparative, showing the geometries optimized are close in structure and energy. The difference lies in the choice of single point calculation methods. The solvent choice is also different. This work is in toluene (non-polar), and work by Gärtner et al. is in diethyl ether (polar).

4. The “Anti” and “Syn” structures of the borylene complexes.

Figure S3 presents the structural features of the parent borylene species [(CAAC)(Dur)BBr_2] (**1**), the N_2-bridged borylene species (**4**). The orientation of the Dip group relative to the Dur group

can result in either an “Anti” or “Syn” configuration. The Anti isomer, **1a** has the Dip and the Dur opposite to each other, while the Syn isomer **1b** has them stacked together.

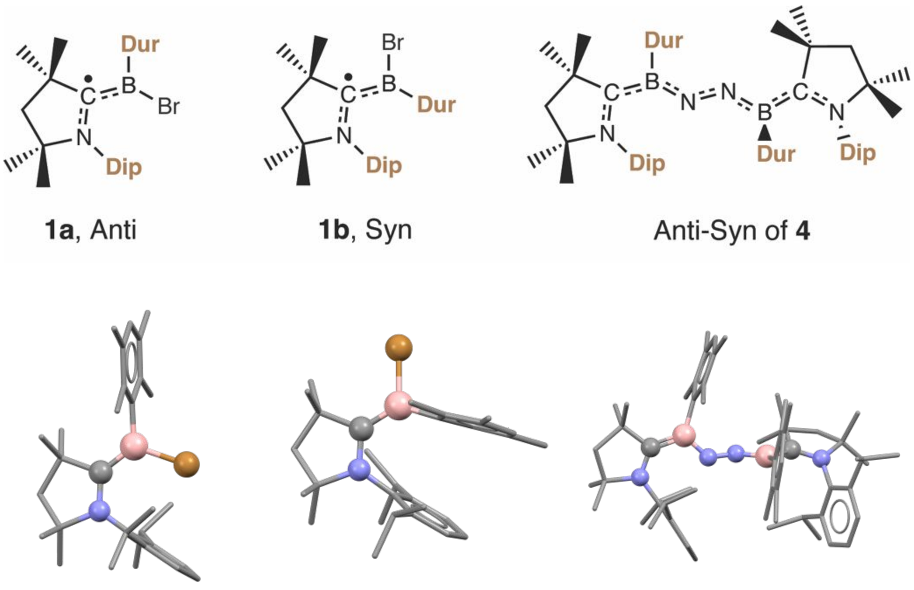

1a, Anti **1b**, Syn Anti-Syn of **4**

Figure S3. The “Anti” (**1a**) and “Syn” (**1b**) conformers of **1**; and **4** in “Anti-Syn” geometry, optimized by DFT. Hydrogens are omitted for clarity.

The Gibbs free energy difference (ΔG) between the **1a** and **1b** conformers is 15.1 kcal/mol, with the Anti conformer **1a** being more stable both computationally and experimentally. The transition state barrier ($\Delta G^{\ddagger}$) to convert **1b** into **1a** is 8.7 kcal/mol. In truncated models (**1_trun**), where all the methyl and iso-propyl groups of **1** are replaced by hydrogen atoms, the Gibbs free energy of the anti-conformer (**1a_trun**) is only 1.8 kcal/mol lower than its Syn isomer (**1b_trun**), This difference is substantially smaller than the 15.1 kcal/mol energy gap between the full models **1a** and **1b**, highlighting the critical role of steric hindrance in determining molecular conformations. Upon reduction, **1** loses its Br atom and yields **2**, featuring a linear C^{CAAC}-B-C^{Dur} structure regardless of whether it transitions from **1a** or **1b**.

During the reduction process, the dinitrogen borylene complexes can adopt structures comprising two halves, with possible Anti-Anti, Anti-Syn, or Syn-Syn conformation. A comprehensive conformational search for species **4** was performed, with results compiled in **Table S2**. Among all three conformations, species with Anti-Syn geometries generally exhibit the highest thermodynamic stability (in agreement with the isolated crystal structures), followed by those with Syn-Syn and Anti-Anti conformations. Given that these complexes feature four substituted benzene rings (two Dip and two Dur groups), various non-covalent interactions including π/π stacking[2–4] and CH/π interaction[5–8] significantly influence their stability. As only the Anti-Syn and Syn-Syn conformations were experimentally observed, these two conformations were specifically investigated for each species (**Table S3**). In the energy diagrams presented in this paper, only the thermodynamically most stable structure for each species is shown.

It is also noted that the dinitrogen borylene complexes consist of over 150 atoms and are generally soft and flexible, which allows for easy geometrical changes. The rotation of the t-butyl and methyl groups, as well as the bending of the five-membered rings, although minor, all contribute to the variations in energy.

4.1. The comparison for species 4 in different conformations.

Table S2. The relative electronic energy (E_e) and structural information of local minima for **4**, with Anti-Syn, Syn-Syn and Anti-Anti conformations. The experimentally isolated crystal structure is highlighted in **bold** for comparison.

Name	Confor-mation	Relative E_e in kcal/mol	N2-N3 bond distance/Å	B1-N2-N3-B2 Dihedral angle/°	B1-C1-N1-C3 Dihedral angle/°	B2-C2-N2-C4 Dihedral angle/°
4_AA1	Anti-Anti	12.75	1.27	123.5	-160.7	-161
4_AA2		11.36	1.25	78.2	173.4	173.4
4_AA3		10.69	1.25	-103.9	174.7	172.2
4_AA4		10.04	1.25	100.7	-179.1	-179
4_AA5		5.86	1.24	100.7	-141.7	-142
4_SS1	Syn-Syn	7.95	1.25	-107.6	-53.3	46.5
4_SS2		7.11	1.25	108.9	54.2	-49
4_SS3		5.36	1.23	97.1	-46.8	-46.8
4_SS4		5.17	1.23	-97.0	46.7	46.7
4_SS5		3.21	1.24	-111.7	-51.4	-51.4
4_SS6		3.04	1.24	111.7	51.3	51.3
4_AS1	Anti-Syn	5.17	1.25	-115.9	174.2	-53.6
4_AS2		4.95	1.23	95.4	179.5	-48.9
4_AS3		3.39	1.22	86.0	170.8	48.8
4_AS4		0.63	1.23	99.3	-173.9	-50.4
4_AS5		0.63	1.23	-99.3	173.9	50.4
4_AS6		0.04	1.24	-112.1	171.7	-52.4
4_AS7		0.00	1.24	110.7	179.2	52.8
**4_Crytal**	**Anti-Syn**	**---**	**1.25**	**113.3**	**176.8**	**51.8**

4.2. The isomers involved for each species in the reaction

Table S3. Calculated relative Gibbs free energy within each species in the reaction, with thermodynamically most stable isomer as 0.

Species and its critical structure	Conformation	Relative Gibbs Free Energy (ΔG, kcal/mol)	Structure
**1** N-B-Br	**Anti-Syn***	0.0	
	Syn-Syn	15.1	
**3** N-B-N_2	**Anti-Syn***	0.0	
	Syn-Syn	5.3	
**4** BNNB^0 ([0H]^0)	**Anti-Syn***	0.0	
	Syn-Syn	2.3	
**4$^-$** BNNB$^-$ ([0H]$^-$)	Anti-Syn	2.5	
	**Syn-Syn**	0.0	
**5^{2-}** BNNB^{2-} ([0H]$^{2-}$)	Anti-Syn	3.0	
	**Syn-Syn***	0.0	

<table>
<tr><td rowspan="3">[1H]$^{+}$</td><td>1H^{+}_AH</td><td>0.0</td><td rowspan="12">[1H$^{+/0/-/2-}$_AH]:
Anti-Syn structure with H on the Anti side of N atom

[1H$^{+/0/-/2-}$_SH]:
Anti-Syn structure with H on the Syn side of N atom

[1H$^{+/0/-/2-}$_SS]:
Syn-Syn structure with H on the N atom</td></tr>
<tr><td>1H^{+}_SH</td><td>1.6</td></tr>
<tr><td>1H^{+}_SS</td><td>1.1</td></tr>
<tr><td rowspan="3">[1H]^{0}</td><td>1H^{0}_AH</td><td>0.2</td></tr>
<tr><td>1H^{0}_SH</td><td>1.3</td></tr>
<tr><td>1H^{0}_SS</td><td>0.0</td></tr>
<tr><td rowspan="3">[1H]$^{-}$</td><td>1H^{-}_AH</td><td>1.7</td></tr>
<tr><td>1H^{-}_SH</td><td>1.6</td></tr>
<tr><td>1H^{-}_SS</td><td>0.0</td></tr>
<tr><td rowspan="3">[1H]$^{2-}$</td><td>1H^{2-}_AH</td><td>0.6</td></tr>
<tr><td>1H^{2-}_SH</td><td>0.9</td></tr>
<tr><td>1H^{2-}_SS</td><td>0.0</td></tr>
<tr><td rowspan="2">[2H]$^{+}$</td><td>Anti-Syn</td><td>0.0</td><td rowspan="8">Anti-Syn:
Anti-Syn structure with H on both sides of N atom

Syn-Syn:
Syn-Syn structure with H on both sides of N atom</td></tr>
<tr><td>Syn-Syn</td><td>2.5</td></tr>
<tr><td rowspan="2">[2H]^{0}</td><td>Anti-Syn*</td><td>0.0</td></tr>
<tr><td>Syn-Syn</td><td>3.6</td></tr>
<tr><td rowspan="2">[2H]$^{-}$</td><td>Anti-Syn</td><td>0.0</td></tr>
<tr><td>Syn-Syn</td><td>1.7</td></tr>
<tr><td rowspan="2">[2H]$^{2-}$</td><td>Anti-Syn</td><td>0.0</td></tr>
<tr><td>Syn-Syn</td><td>3.2</td></tr>
</table>

Footnote: The ***** represent the species that has been experimentally synthesized (with the counterions). The most thermodynamic stable isomer is in **bold.**

Table S4. Electronic energy comparison between isomers that's protonated on B and/or N atoms within each species.

Species	Critical structure	Relative Electronic Energy (ΔE_e, kcal/mol)	Note
**$[1H]^+$**	**AH**	0.0	The comparison is restricted to Anti-Syn structured isomers that are singly protonated. Protonation on the boron atom occurs exclusively at the Anti side.<br><br>AH: Anti-Syn structure with H on Anti-side N atom<br>SH: Anti-Syn structure with H on Syn-side N atom<br>BH: Anti-Syn structure with H on Anti-side boron atom
	BH	17.6	
**$[1H]^0$**	**AH**	0.0	
	BH	33.5	
**$[1H]^-$**	**AH**	0.0	
	BH	35.3	
**$[1H]^{2-}$**	**SH**	0.0	
	BH	22.2	
**$[2H]^+$**	**NH-NH**	0.0	The comparison is restricted to Anti-Syn structured isomers that are doubly protonated. Protonation on the boron atom occurs exclusively at the Anti side.
	NH_2	33.5	
	BH-NH	29.3	
	BH-N-NH	35.6	
**$[2H]^0$**	**NH-NH**	0.0	

	NH_2	40.2	NH_2: species where hydrogenation occurs on the same nitrogen atom at the Anti side.
	BH-NH	18.9	
	BH-N-NH	32.0	
**$[2H]^-$**	**NH-NH**	0.0	
	NH_2	21.2	
	BH-NH	20.9	
	BH-N-NH	30.5	
**$[2H]^{2-}$**	**NH-NH**	0.0	
	NH_2	28.1	
	BH-NH	25.1	
	BH-N-NH	33.6	
**$[3H]^+$**	BH-NH-NH	0.0	The comparison is restricted to Anti-Syn structured isomers that are triply protonated. Protonation on the boron atom occurs exclusively at the Anti side.<br><br>NH_2-NH: species where there are two hydrogens on the same nitrogen atom at the Anti side, one hydrogen on the nitrogen atom at the Syn side.
	NH_2-NH	19.0	
**$[3H]^0$**	BH-NH-NH	0.0	
	NH_2-NH	12.1	
**$[3H]^-$**	BH-NH-NH	6.6	
	NH_2-NH	0.0	
**$[4H]^+$**	NH_2-NH_2	89.0	The comparison is restricted to Anti-Syn structured isomers that are quadruply
	BH-NH-NH_2	55.4	

	BH-NH-NH-BH	48.2	protonated. Protonation on the boron atom occurs exclusively at the Anti side.
	BH-NH_2-NH	37.7	
	NH_2---NH_2 (NN bond breaks)	0.0	
**[4H]^0**	BH-NH-NH-BH	62.7	
	BH-NH-NH_2	58.7	
	BH-NH_2-NH	52.9	
	NH_2---NH_2 (NN bond breaks)	0.0	

4.3. Isomerization of hydrogen transfer from boron to nitrogen site.

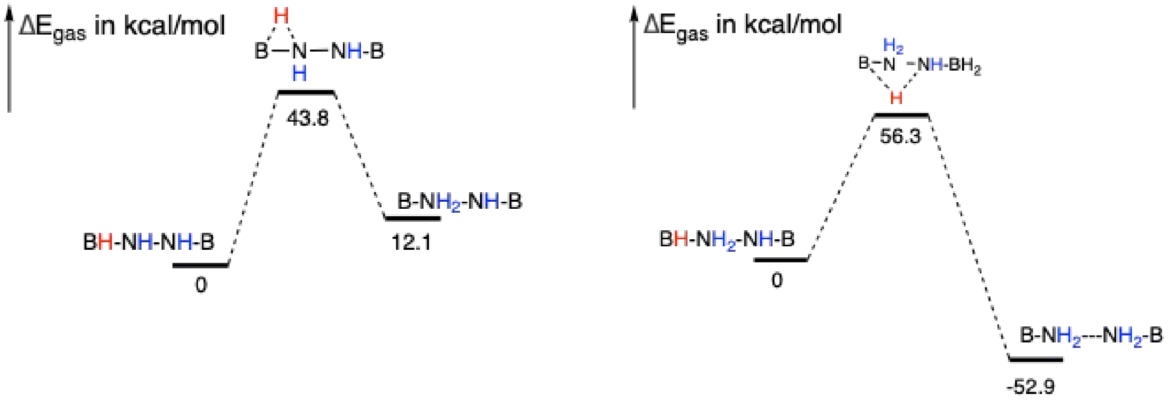

Figure S4. The gas phase electronic energy change for hydrogen transition from boron to nitrogen site: **[3H]^0** isomerization (left), and **[4H]^0** isomerization (right). (Energy not to scale).

5. Optimized structures and spin density

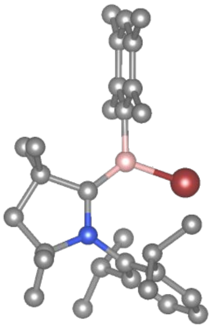<br>**1(1a)**	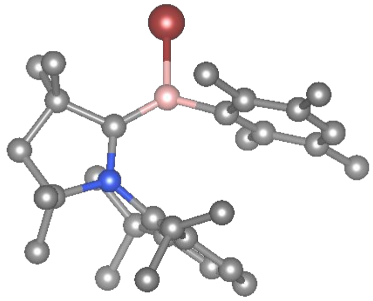<br>**1b**
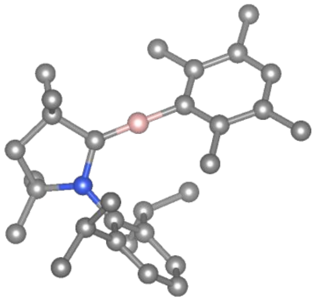<br>**2**	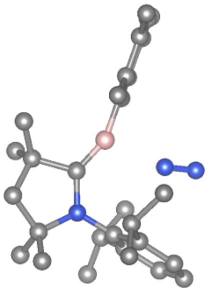<br>**$TS_{2\text{-}3}$**
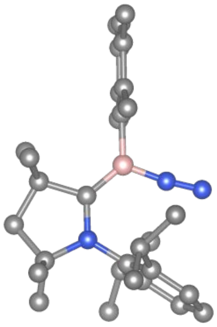<br>**3**	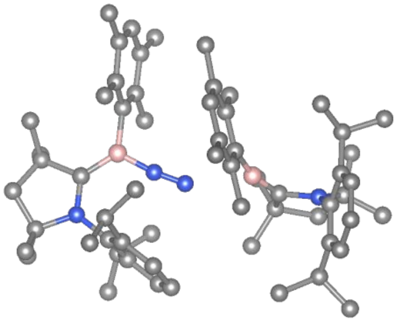<br>**$TS_{3\text{-}4}$**

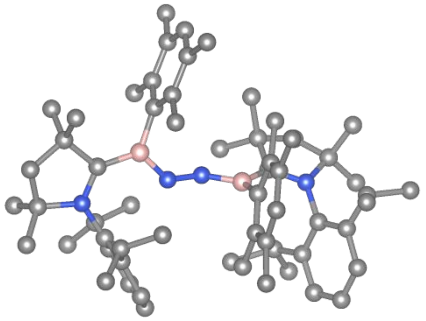

4

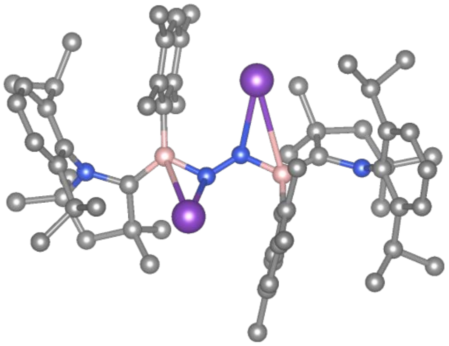

$^{1}5$

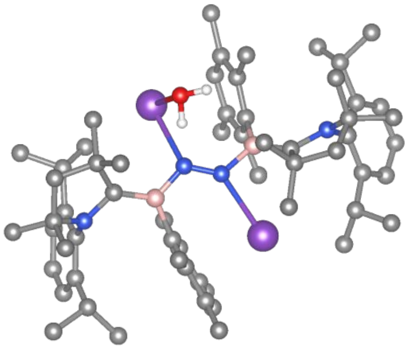

$^{1}5_{H2O}$

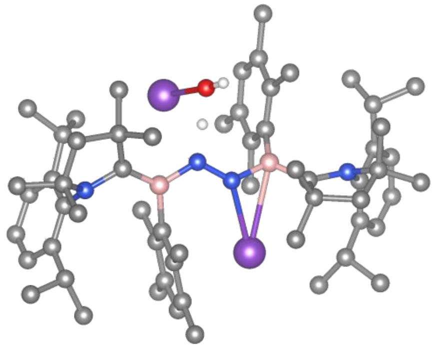

$^{1}TS_{5\text{-}6}$

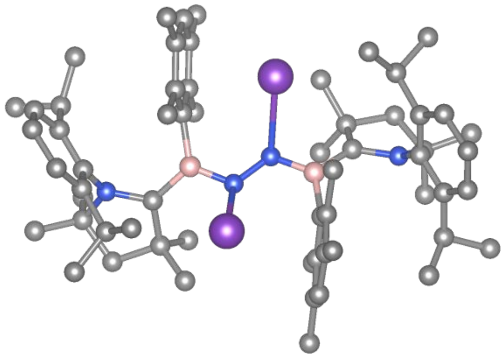

$^{3}5$

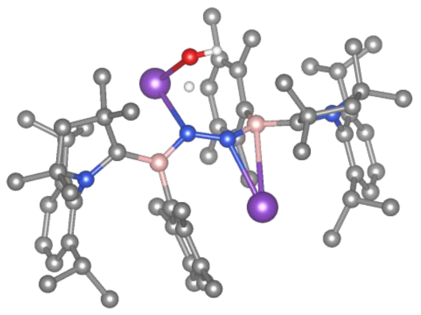<br>$^{3}\mathbf{5}_{\mathbf{H2O}}$	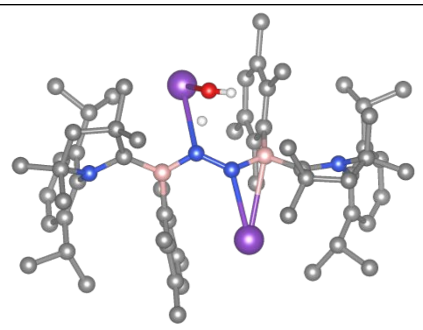<br>$^{3}\mathbf{TS}_{\mathbf{5\text{-}6}}$
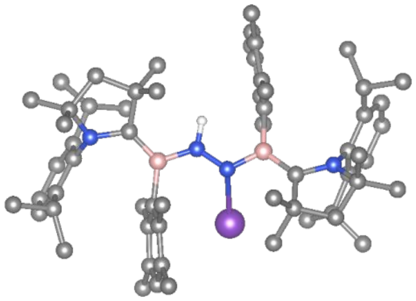<br>$^{1}\mathbf{6}$	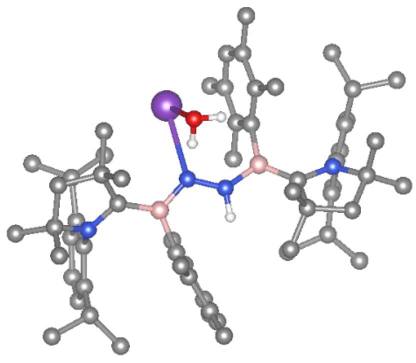<br>$^{1}\mathbf{6}_{\mathbf{H2O}}$
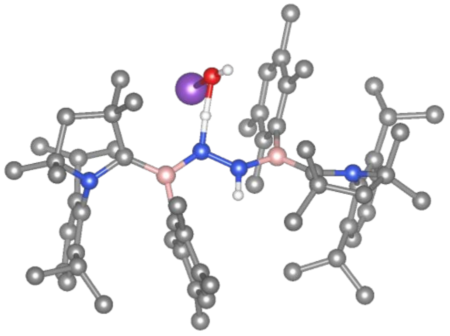<br>$^{1}\mathbf{TS}_{\mathbf{6\text{-}7}}$	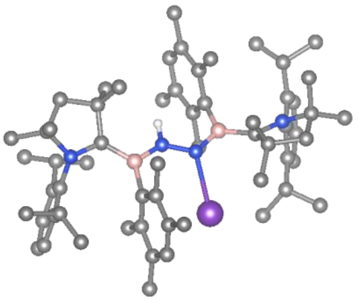<br>$^{3}\mathbf{6}$

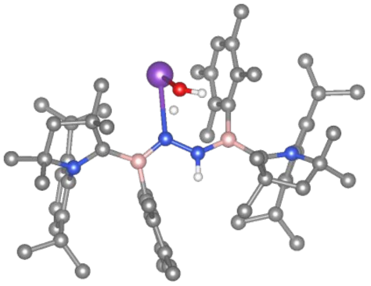<br>**$^{3}6_{H2O}$**	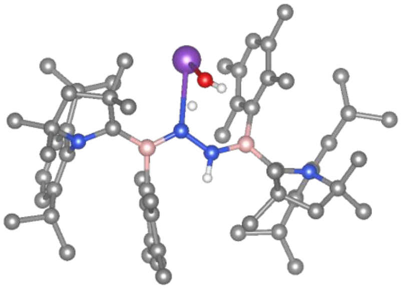<br>**$^{3}TS_{6-7}$**
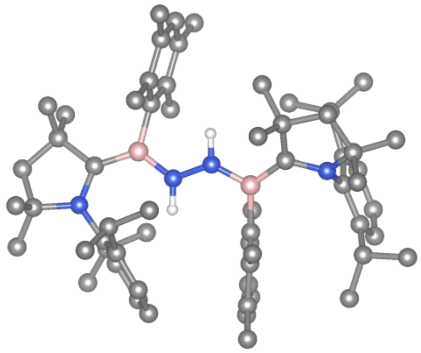<br>**$^{1}7$**	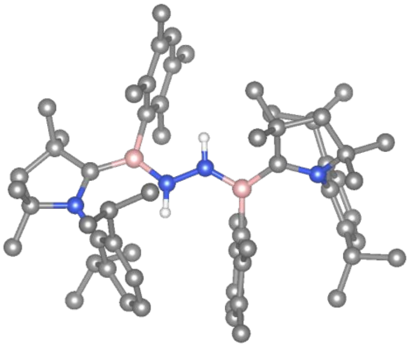<br>**$^{3}7$**
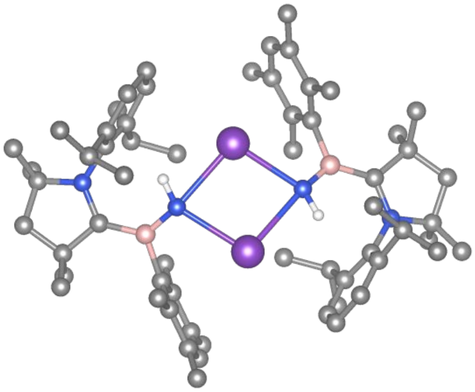<br>**8_dimer**	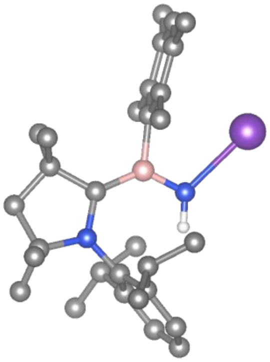<br>**8**

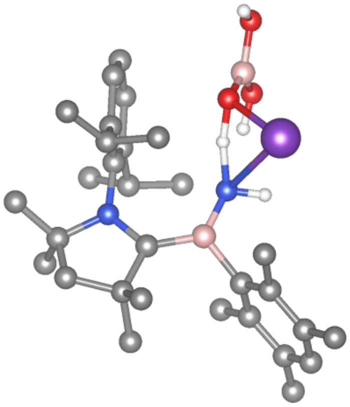

TS$_{8\text{-}9}$

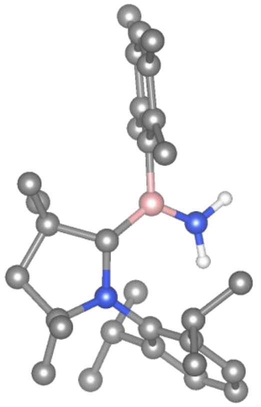

9

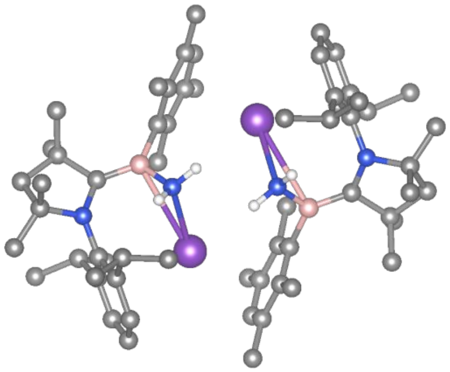

10_dimer

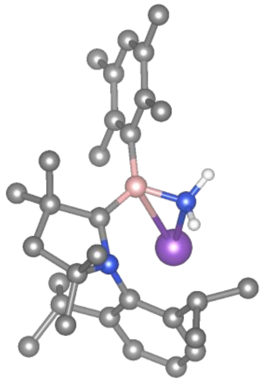

10

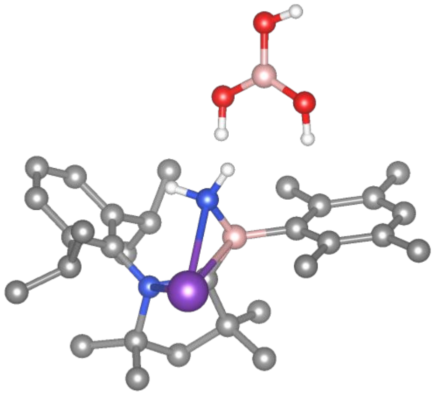

TS$_{10\text{-}11}$

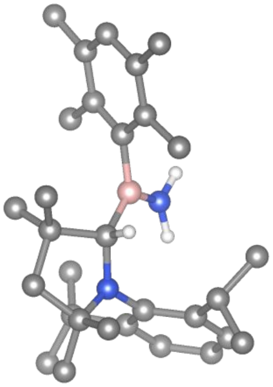

11

Figure S5. DFT optimized structures of all intermediates during borylene mediated N_2 reduction reaction. Hydrogens are omitted unless on the B or N atoms.

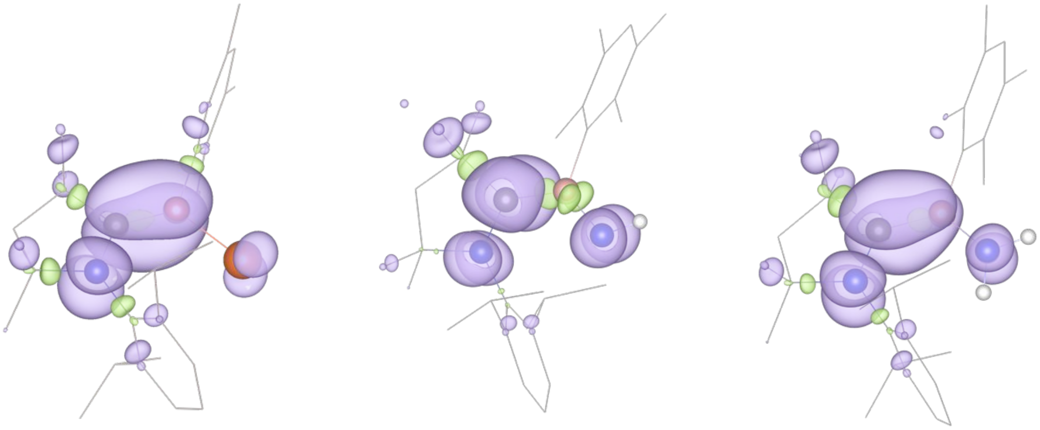

1 **8** **9**

Figure S6. Spin density of **1**, **8**, **9** (isovalue = 0.0004). Hydrogens are omitted for clarity.

6. References

(1) Marenich, A. V.; Cramer, C. J.; Truhlar, D. G. Universal Solvation Model Based on Solute Electron Density and on a Continuum Model of the Solvent Defined by the Bulk Dielectric Constant and Atomic Surface Tensions. *J. Phys. Chem. B* **2009**, *113* (18), 6378–6396. https://doi.org/10.1021/jp810292n.

(2) Hunter, C. A.; Sanders, J. K. M. The Nature of .Pi.-.Pi. Interactions. *J. Am. Chem. Soc.* **1990**, *112* (14), 5525–5534. https://doi.org/10.1021/ja00170a016.

(3) Grimme, S. Do Special Noncovalent π–π Stacking Interactions Really Exist? *Angew. Chem. Int. Ed.* **2008**, *47* (18), 3430–3434. https://doi.org/10.1002/anie.200705157.

(4) R. Martinez, C.; L. Iverson, B. Rethinking the Term “Pi-Stacking.” *Chem. Sci.* **2012**, *3* (7), 2191–2201. https://doi.org/10.1039/C2SC20045G.

(5) Nishio, M. CH/π Hydrogen Bonds in Crystals. *CrystEngComm* **2004**, *6* (27), 130–158. https://doi.org/10.1039/B313104A.

(6) Jiang, Y.; Xi, C.; Liu, Y.; Niclós-Gutiérrez, J.; Choquesillo-Lazarte, D. Intramolecular “CH···π(Metal Chelate Ring) Interactions” as Structural Evidence for Metalloaromaticity in Bis(Pyridine-2,6-Diimine)RuII Complexes. *Eur. J. Inorg. Chem.* **2005**, *2005* (8), 1585–1588. https://doi.org/10.1002/ejic.200400864.

(7) Milčić, M. K.; Medaković, V. B.; Zarić, S. D. CH/π Interactions of π-System of Acetylacetonato Chelate Ring: Comparison of CH/π Interactions of Ni(II)-Acetylacetonato Chelate and Benzene Rings. *Inorganica Chim. Acta* **2006**, *359* (13), 4427–4430. https://doi.org/10.1016/j.ica.2006.06.022.

(8) Milčič, M. K.; Medaković, V. B.; Sredojević, D. N.; Juranić, N. O.; Zarić, S. D. Electron Delocalization Mediates the Metal-Dependent Capacity for CH/π Interactions of Acetylacetonato Chelates. *Inorg. Chem.* **2006**, *45* (12), 4755–4763. https://doi.org/10.1021/ic051926g.
